# Supplementary material for: HIF2-driven PTHrP Causes Cachexia and Hypercalcemia in Kidney Cancer: Treatment with HIF2 Inhibitors
Source: bioRxiv. 2025 Sep 11:2025.09.09.675147. Preprint. [Version 1] doi: 10.1101/2025.09.09.675147 (PMC12439952; doi:10.1101/2025.09.09.675147)
Supplement: Supplement 2 [file media-2.pdf]

	HIF2i (N=46)	ICI (N=56)	VEGF TKI (N=49)	Overall (N=151)	P-value
**Age at treatment start**					
Median IQR	66.1 [60.0, 70.1]	61.9 [57.4, 67.4]	62.9 [55.9, 66.3]	63.3 [57.2, 68.0]	0.07
**Sex**					
Female	13 (28.3%)	13 (23.2%)	10 (20.4%)	36 (23.8%)	0.66
Male	33 (71.7%)	43 (76.8%)	39 (79.6%)	115 (76.2%)	
**ECOG at treatment start**					
0	27 (58.7%)	40 (71.4%)	27 (55.1%)	94 (62.3%)	0.31
1	14 (30.4%)	13 (23.2%)	16 (32.7%)	43 (28.5%)	
2	4 (8.7%)	1 (1.8%)	4 (8.2%)	9 (6.0%)	
Missing	1 (2.2%)	2 (3.6%)	2 (4.1%)	5 (3.3%)	
**Best response**					
CR	0 (0%)	4 (7.1%)	1 (2.0%)	5 (3.3%)	0.09
PR	9 (19.6%)	18 (32.1%)	17 (34.7%)	44 (29.1%)	
SD	19 (41.3%)	20 (35.7%)	23 (46.9%)	62 (41.1%)	
PD	15 (32.6%)	11 (19.6%)	7 (14.3%)	33 (21.9%)	
Missing	3 (6.5%)	3 (5.4%)	1 (2.0%)	7 (4.6%)	
***VHL* status**					
Wild-type	4 (8.7%)	5 (8.9%)	6 (12.2%)	15 (9.9%)	0.918
Mutated	9 (19.6%)	8 (14.3%)	11 (22.4%)	28 (18.5%)	
Missing	33 (71.7%)	43 (76.8%)	32 (65.3%)	108 (71.5%)	
**Stage**					
III	0 (0%)	3 (5.4%)	1 (2.0%)	4 (2.6%)	0.23
IV	46 (100%)	53 (94.6%)	48 (98.0%)	147 (97.4%)	
**Line of therapy**					
1	0 (0%)	47 (83.9%)	37 (75.5%)	84 (55.6%)	<0.001
2	5 (10.9%)	4 (7.1%)	9 (18.4%)	18 (11.9%)	
3	13 (28.3%)	2 (3.6%)	3 (6.1%)	18 (11.9%)	
4	13 (28.3%)	2 (3.6%)	0 (0%)	15 (9.9%)	
5+	15 (32.6%)	1 (1.8%)	0 (0%)	16 (10.6%)	
**Corrected calcium at start of therapy (mg/dl)**					
Median IQR	9.62 [9.40, 9.88]	9.43 [9.12, 9.80]	9.52 [9.28, 9.80]	9.54 [9.27, 9.86]	0.24
**BMI at start of therapy (kg/m^2)**					

	HIF2i (N=46)	ICI (N=56)	VEGF TKI (N=49)	Overall (N=151)	P-value
Median IQR	27.5 [24.2, 29.6]	27.7 [24.1, 32.5]	28.6 [25.5, 34.6]	27.7 [24.9, 32.0]	0.08

Table 1: Baseline characteristic of the patients with clear-cell renal cell carcinoma from the Dana-Farber cohort

CR: complete response, ECOG: Eastern Cooperative Oncology Group, HIF2i: HIF-2α inhibitors, ICI: immune checkpoint inhibitors, IQR: interquartile range, PD: progressive disease, PR: partial response, SD: stable disease, VEGF TKI: vascular endothelial growth factor tyrosine kinase inhibitor, *VHL*: Von-Hippel Lindau gene

Statistical comparisons between the 3 treatment groups were performed using the Chi-square test for categorical variables and the Kruskal-Wallis test for continuous variables.
