# Supplementary material for: HIF2-driven PTHrP Causes Cachexia and Hypercalcemia in Kidney Cancer: Treatment with HIF2 Inhibitors
Source: bioRxiv. 2025 Sep 11:2025.09.09.675147. Preprint. [Version 1] doi: 10.1101/2025.09.09.675147 (PMC12439952; doi:10.1101/2025.09.09.675147)
Supplement: Supplement 3 [file media-3.pdf]

	Low baseline PTHrP (≤ Q1) (N=12)	Mid baseline PTHrP (Q1- Q3) (N=22)	High baseline PTHrP (> Q3) (N=11)	Total Evaluable (N=45)
**Age at treatment start (years)**				
Median [min-max]	58 [47-78]	67.5 [38-87]	68 [53-74]	64 [38-87]
**Sex**				
Female	2 (16.7%)	9 (40.9%)	4 (36.4%)	15 (33.3%)
Male	10 (83.3%)	13 (59.1%)	7 (63.6%)	30 (66.7%)
**ECOG at treatment start**				
0	6 (50.0%)	10 (45.5%)	3 (27.3%)	19 (42.2%)
1	6 (50.0%)	11 (50.0%)	7 (63.6%)	24 (53.3%)
2	0 (0.0%)	1 (4.5%)	0 (0.0%)	1 (2.2%)
Missing	0 (0.0%)	0 (0.0%)	1 (9.1%)	1 (2.2%)
**Best response**				
CR	0 (0.0%)	0 (0.0%)	0 (0.0%)	0 (0.0%)
PR	1 (8.3%)	9 (40.9%)	2 (18.2%)	12 (26.7%)
SD	6 (50.0%)	10 (45.5%)	4 (36.4%)	20 (44.4%)
PD	5 (41.7%)	3 (13.6%)	4 (36.4%)	12 (26.7%)
Missing	0 (0.0%)	0 (0.0%)	1 (9.1%)	1 (2.2%)
**Stage**				
IA	1 (8.3%)	0 (0.0%)	0 (0.0%)	1 (2.2%)
IB	0 (0.0%)	1 (4.5%)	0 (0.0%)	1 (2.2%)
IIA	0 (0.0%)	5 (22.7%)	0 (0.0%)	5 (11.1%)
IIB	0 (0.0%)	0 (0.0%)	0 (0.0%)	0 (0.0%)
IIIA	6 (50.0%)	6 (27.3%)	4 (36.4%)	16 (35.6%)
IIIB	1 (8.3%)	2 (9.1%)	0 (0.0%)	3 (6.7%)
IV	2 (16.7%)	4 (18.2%)	7 (63.6%)	13 (28.9%)
Other	0 (0.0%)	3 (13.6%)	0 (0.0%)	3 (6.7%)
Missing	2 (16.7%)	1 (4.5%)	0 (0.0%)	3 (6.7%)
**Line of therapy**				
1	3 (25.0%)	3 (13.6%)	0 (0.0%)	6 (13.3%)
2	3 (25.0%)	3 (13.6%)	4 (36.4%)	10 (22.2%)
3	2 (16.7%)	8 (36.4%)	3 (27.3%)	13 (28.9%)

	Low baseline PTHrP (≤ Q1) (N=12)	Mid baseline PTHrP (Q1- Q3) (N=22)	High baseline PTHrP (> Q3) (N=11)	Total Evaluable (N=45)
4	2 (16.7%)	2 (9.1%)	2 (18.2%)	6 (13.3%)
5	0 (0.0%)	2 (9.1%)	1 (9.1%)	3 (6.7%)
6+	2 (16.7%)	4 (18.2%)	1 (9.1%)	7 (15.6%)
**Corrected calcium at start of therapy (mmol/L)**				
Median [min-max]	2.29 [2.12-2.62]	2.38 [2.07-2.62]	2.45 [2.26-3.45]	2.40 [2.07-3.45]
**Body weight at start of therapy (kg)**				
Median [min-max]	97.6 [61.6-127]	75.1 [49.6-117]	84.6 [60.3-94.3]	81.8 [49.6-127]

Table 2: Baseline characteristic of the patients with clear-cell renal cell carcinoma from the Nikang cohort
CR: complete response, ECOG: Eastern Cooperative Oncology Group, PD: progressive disease, PR: partial response, PTHrP: parathyroid hormone-related protein, Q1: first quartile, Q2: second quartile, Q3: third quartile, SD: stable disease.
